# Alternative Disinfection in Hot Water Networks: Persistence and Antimicrobial Efficacy of Silver-Stabilized Hydrogen Peroxide for *Legionella pneumophila* Control

**DOI:** 10.1021/acsestwater.6c00121

**Published:** 2026-05-23

**Authors:** Nate Clark, Lynda H. McCarthy, Steven N. Liss

**Affiliations:** † 7984 Toronto Metropolitan University, Toronto, Ontario M5B 2K3, Canada; ‡ Queen’s University, Kingston, Ontario K7L 3N6, Canada; § Stellenbosch University, Stellenbosch, Western Cape 7602, South Africa

**Keywords:** Legionella pneumophila, Hot water network, Silver-stabilized hydrogen peroxide, Premise plumbing, Oxidant decay, chlorine stability

## Abstract

Engineered water systems operate under diverse chemical and physical conditions that influence disinfection efficiency. Hot water networks (HWNs) exemplify this issue, as thermal control is costly and ineffective in cooled sections, and chlorine degrades rapidly at elevated temperatures. Consequently, HWNs remain challenging for controlling pathogens such as *Legionella pneumophila*. Sustained HWN protection requires a thermally resilient, persistent disinfectant. This study evaluated the stability and efficacy of silver-stabilized hydrogen peroxide (SSHP) in ultrapure, synthetic, and chloraminated municipal tap waters incubated at elevated temperatures for up to 72 h. Culture-based enumeration showed that SSHP antimicrobial efficacy increased with temperature, achieving >2.4-log reductions of *L. pneumophila* within 5 min at 55 °C. SSHP retained full biocidal activity after 72 h at 60 °C in synthetic tap water, whereas free chlorine lost antimicrobial activity after 24 h. In municipal waters, total and free chlorine decayed below recommended thresholds within 24–40 h at 60 °C, whereas SSHP retained effective residuals after 72 h. Although these controlled experiments cannot capture real-system complexity, SSHP persisted under thermal conditions that accelerated chlorine decay, supporting its use as a resilient HWN disinfectant. This work demonstrates how system-specific evaluation of disinfectants can inform improved strategies for managing microbial risks in water systems.

## Graphical abstract

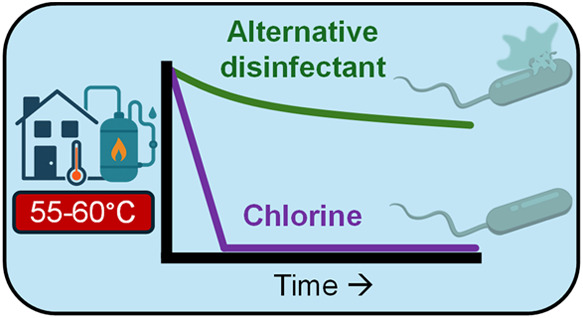

## 1 Introduction

Drinking water systems operate under complex and dynamic conditions that drive fine-scale variation in water quality. To manage microbial risks within this variability, most utilities rely heavily—often exclusively—on chlorine for primary and secondary disinfection. Chlorine’s broad-spectrum antimicrobial activity has made it indispensable as a disinfectant, but its high reactivity poses an enduring operational challenge: sustaining protective residuals throughout distribution systems and premise plumbing.^1^ This fundamental trade-off between disinfection strength and chemical stability continues to shape the operation of drinking water systems and carries critical implications for public health.

*Legionella pneumophila* is an opportunistic waterborne pathogen and the primary etiologic agent responsible for Legionnaires’ disease (LD), a severe and potentially fatal form of pneumonia. Reported cases of LD have increased substantially over the past decade across North America, Europe, Asia, and Oceania, underscoring the growing global challenge of controlling *Legionella* in engineered water systems.^2–10^ In many regions, LD now represents one of the most significant waterborne diseases in terms of hospitalization burden and mortality, placing increasing pressure on public health systems and infrastructure management.^11,12^ This burden is closely tied to the persistence and amplification of *L. pneumophila* within hot water networks (HWNs), which are frequently implicated in LD outbreaks.^13,14^

HWNs represent a particularly challenging subset of premise plumbing for disinfection, where conditions differ markedly from cold water systems. Operating at temperatures as high as 55–60 °C and often subject to extended stagnation, HWNs accelerate disinfectant decay through the combined effects of elevated temperature, long residence times, and interactions with corrosion products and natural organic matter (NOM).^1,15–17^ For example, Liu and Reckhow^16^ showed that chlorine residuals in filtered surface water declined rapidly when samples were heated to 55 °C after a period of stagnation. In their study, water was first held under stagnant conditions and then heated: after 6 h of stagnation, chlorine fell below detection within 3 h of heating, whereas after 24 h of stagnation, this occurred within 30 min. This rapid depletion raises concern that chlorine concentrations in HWNs may frequently fall below the levels required to effectively suppress microbial growth.^16,18,19^ As residuals diminish, the risk of opportunistic pathogen proliferation increases.^18–20^ Elevated temperature also accelerates the formation of chlorine’s disinfection byproducts, several of which have been associated with adverse health outcomes, including bladder cancer, although their formation depends on system-specific factors such as water chemistry and precursor availability.^21–24^ These issues have prompted questions about whether chlorine alone can reliably and safely maintain microbial control in warm, hot, or stagnant water environments.^1^

To counteract disinfectant decay, public health and regulatory organizations commonly recommend thermal control strategies, such as maintaining storage temperatures ≥ 60 °C and delivering hot water at ≥ 50–55 °C at distal points.^25–28^ While thermal control is widely regarded as an effective strategy, sustaining 60 °C throughout plumbing systems is energy intensive; hot water generation is among the largest contributors to household energy use globally, second only to space heating.^29–32^ Even modest reductions in set points (5–6 °C) could yield substantial savings—for example, an estimated $210–$350 million annually for Canadian residents (Table S1)—while also reducing scalding risk, environmental impacts, and infrastructural strain. Complicating matters further, even at nominal 60 °C set points, HWNs often contain segments in the 35–45 °C range that support the growth of some opportunistic pathogens, such as *L. pneumophila*.^19,33^ Thus, even when paired with chlorine, thermal treatment may not fully suppress opportunistic pathogens and may contribute to the emergence of thermotolerant strains.^34–36^ Lowering HWN temperatures could provide substantial energy, safety, and sustainability benefits—but this is only feasible if paired with a disinfectant that is thermally stable and effective under realistic hot water conditions.^36,37^

Taken together, these challenges reflect a broader principle that extends beyond HWNs: disinfectant performance is shaped not only by intrinsic antimicrobial potency, but also by system-specific thermal, chemical, and hydraulic stressors. Given the spatial and temporal variation in these stressors across engineered water systems, no single disinfectant is likely to perform optimally in all contexts. Ensuring effective and sustainable microbial control therefore depends not only on selecting a potent oxidant, but also on identifying one whose properties align with the constraints of the system in which it must operate. Evaluating disinfectants under system-specific conditions can reveal where they excel and help shift practice away from a “silver bullet” solution toward diversified, context-dependent strategies. HWNs provide a compelling test case for this systems-level perspective, as they impose extreme, but illustrative, constraints on disinfectant stability, reactivity, and effectiveness.

To demonstrate how this systems-level perspective can inform disinfectant selection, we evaluated the performance of an alternative oxidant under the challenging thermal and chemical conditions characteristic of HWNs. Silver-stabilized hydrogen peroxide (SSHP) is a formulation of hydrogen peroxide and ionic silver that has been employed as an alternative disinfectant in water systems. When applied at residual concentrations, the silver present in SSHP remains well below established regulatory limits (e.g., ≤ 0.1 mg/L),^38^ and its use is associated with substantially reduced formation of regulated disinfection byproducts.^39^ While hydrogen peroxide-based disinfection can generate low levels of aldehydes or ketones as byproducts, their formation is condition-dependent and they are not considered a significant public health concern at concentrations relevant to drinking water treatment. Field, pilot, and full-scale studies indicate that SSHP can (i) sustain measurable residuals in distribution systems, (ii) control microbial growth in premise plumbing and drinking water networks, and (iii) serve as a targeted intervention for *Legionella* spp. in HWNs.^34,39,40^ SSHP also exhibits antimicrobial synergy relative to hydrogen peroxide alone,^41–46^ appears less susceptible than chlorine to NOM-driven decomposition,^39,41^ and shows increased antimicrobial activity at elevated temperatures (up to 40 °C).^39,43,45^ Despite these attributes, SSHP’s stability, decay kinetics, and antimicrobial activity under high-temperature, high-demand conditions remain poorly characterized.

In this work, we examine the thermal stability and antimicrobial activity of SSHP, comparing its performance to chlorine under HWN-relevant conditions in ultrapure, synthetic, and municipal waters. This study evaluates SSHP under stressors that routinely limit chlorine persistence in HWNs—elevated temperatures, stagnation, and matrix-driven oxidant demand—and provides an initial indication of whether alternative disinfectants might eventually support safe operation at moderately lower temperatures while maintaining control of pathogens, such as *L. pneumophila*. Although this study focuses on bulk water (i.e., in the absence of pipe surfaces and adhered biofilms), the conditions applied here capture several key factors that strongly influence residual maintenance in operational systems. By pairing decay measurements with assessments of antimicrobial efficacy, this study clarifies how oxidant properties and system stressors interact to shape residual performance. More broadly, systematic evaluations of disinfectants under system-specific constraints, such as the approach used in this study, can generate the evidence needed to reassess operating practices and advance toward more resilient and sustainable approaches to water system disinfection.

## 2 Materials and Methods

### 2.1 Media, Material, and Culture Preparation

A culture of *L. pneumophila* (serotype 1, subspecies *pneumophila*), of the Philadelphia-1 strain was obtained from Cedarlane Laboratories (ATCC 33152). This strain was originally isolated from the lung tissue of a person infected with a fatal case of Legionnaires’ disease in Philadelphia, 1976.^47^ *L. pneumophila* cultures were grown either on buffered charcoal yeast extract agar (BCYE-α; 1000 mL of Milli-Q ultrapure water, 17.0 g of agar, 10.0 g of yeast extract, 10.0 g of *N*-(2-acetamido)-2-animoethanesulfonic acid (ACES) buffer, 2.0 g of activated charcoal, 1.0 g of α-ketoglutaric acid, 0.40 g of l-cysteine hydrochloride monohydrate, and 0.25 g of ferric pyrophosphate)^48^ or in buffered yeast extract broth (BYEB; BCYE-α without agar and charcoal). To account for *L. pneumophila*’s sensitivity to sodium, the pH of the medium was adjusted to 6.9 ± 0.2 using 1 M potassium hydroxide. *L. pneumophila* cultures were grown for 48 h at 37 °C in BYEB, corresponding to the postexponential phase as determined by growth curves. Stock cultures of *L. pneumophila* were stored at −80 °C and suspended in BYEB containing 30% glycerol.

Reagent grade sodium hypochlorite (NaOCl) was purchased as a 5.0% stock solution (Sigma-Aldrich, St. Louis, Missouri, United States of America). SSHP was supplied as a stock solution containing 19.9% hydrogen peroxide and 0.013% silver (Huwa-San DW TR20; SanEcoTec Ltd., Ottawa, Ontario, Canada). Free and total chlorine concentrations were assessed via the *N*,*N*-diethyl-p-phenylenediamine method (Hach Methods: 10245, 10069, and 8167; Ranges: 0.05 to 4.00 mg/L, 0.1 to 10 mg/L, and 0.02 to 2.00 mg/L Cl_2_, respectively) using a DR900 colorimeter (Hach Company, Colorado, United States of America). The hydrogen peroxide concentration in SSHP solutions was assessed via the titanium oxide oxalate method (Range: 0.05 to 100 mg/L).^49^ Throughout this study, the concentration of SSHP was represented as the concentration of hydrogen peroxide in the solution, as others have determined that it is the active ingredient in SSHP and that the silver ions have a negligible inhibitory effect at residual concentrations.^46^

A synthetic tap water (SYTW) was used in this study because it (i) had a known composition, (ii) allowed for consistent results free of batch/sampling effects, and (iii) was not previously treated to remove chlorine demand. To approximate municipal drinking water chemistry in a controlled and reproducible manner, the SYTW was created using the 2021 City of Toronto “Drinking Water Quality Annual Analysis Summary Reports” as a guide.^50^ The finished SYTW contained the inorganic components of Toronto drinking water with reported concentrations of 1 μg/L or above (Table S2). Depending on the context of the experiment, SYTW was created (i) without any organics, (ii) with humic acid as the organic source, or (iii) with tannic acid as the organic source.

Prior to each experiment, all glassware was treated for 24 h with 1% solutions of either NaOCl or SSHP to remove their demand. The glassware was then rinsed five times with Milli-Q ultrapure water before then being baked in an oven for several hours.

All experiments were conducted using independent biological replicates (*n* ≥ 3), with technical triplicates used for plating-based enumeration and decay assays. The number of biological replicates for each experiment is indicated in the corresponding figure captions.

### 2.2 Kill-Time Assays: Assessing the Impact of Temperature on SSHP Effectiveness

Kill-time assays were conducted to reveal the temperature-dependent antimicrobial effects of SSHP on planktonic *L. pneumophila* for up to 60 min. Prior to each experiment, postexponential cultures were centrifuged (3000*g*, 5 min, 4 °C) and washed twice in fresh SYTW to remove residual growth medium. The absorbance of the bacterial suspensions at 600 nm (OD_600_) was then measured using a Thermo Scientific Multiskan GO spectrophotometer (Thermo Fisher Scientific Inc., Massachusetts, United States). Cultures were standardized to an OD_600_ of 0.15 (∼1 × 10^8^ colony forming units [CFU/mL]) and subsequently diluted in SYTW to achieve a starting concentration of ∼7 × 10^4^ CFU/mL. Initial cell densities were verified by plating at time zero, with control samples from the same culture plated to confirm CFU/mL estimates. At the beginning of the experiment, SSHP was added to achieve a final concentration of 20 mg/L. For each trial, a second culture was used as a control and was exposed to the same temperatures for the same duration, without disinfectant. Temperature was maintained at 35, 45, or 55 °C by placing the cultures in a heating block submerged in a hot water bath. Throughout each experiment, temperature was monitored via two submerged glass thermometers.

At fixed intervals, 1 mL of culture was removed and immediately neutralized by dilution in 4 °C, filter-sterilized 100 mM phosphate buffer (pH 7.5) containing 1 g/L protease peptone and 0.667 g/L of catalase from bovine liver (Sigma-Aldrich, St. Louis, Missouri, United States of America). Following vortexing, samples were serially diluted in the same neutralization buffer and allowed to sit for 2–3 min before filter-sterilized sodium thioglycolate was added to achieve a final concentration of 1 g/L for silver complexation. This neutralization strategy was selected to ensure rapid and complete quenching of hydrogen peroxide and silver activity, and to prevent continued antimicrobial effects following sampling, as has been observed with thiosulfate-based neutralization approaches.^41^ These dilutions were plated in triplicate on BCYE-α plates, with 100–200 μL plated depending on dilution to ensure countable colony numbers. Plates were incubated at 37 °C for 5–7 days prior to colony enumeration. Plates with between 30 and 300 colonies were counted to estimate viable cell concentration in CFU/mL. Plated volumes were accounted for in CFU/mL calculations. Detection limits were determined based on plated volume and dilution factor, corresponding to a minimum quantifiable threshold of 30 CFU per plate, and varied between assays depending on the initial cell concentration.

### 2.3 Disinfectant Decay Assays

#### 2.3.1 Selecting Incubation Temperature and Disinfectant Concentrations

Solutions of SYTW with free chlorine or SSHP were incubated at 55 or 60 °C to assess their relative stability under high temperature. Incubation at 55 °C was chosen for several reasons. First, temperature stratification and heat loss can mean that parts of a hot water tank fall below 60 °C, particularly in the lower portions or in dead legs, even if the thermostat is set to 60 °C.^33^ Second, multiple public health and regulatory organizations recommend storing water at ≥ 60 °C but delivering it at ≥ 50–55 °C, while using mixing valves to limit scalding risk at the tap.^25–28^ Third, previous studies have selected this temperature to examine chlorine decay in HWNs.^23^ Finally, 55 °C represents a reasonable target for future temperature reduction if balancing between improved sustainability and system performance. However, since 60 °C is still the recommended set point for hot water heaters by several regulatory and professional bodies,^25–28^ it was included in this study for comparison.

Two concentrations were selected for assessing the stability of free chlorine: 2 and 4 mg/L. Both represent “best-case” scenarios for chlorine performance in household HWNs. These values reflect upper-limit concentrations used in treated drinking water based on regulatory standards in North America, where 4 mg/L is the maximum residual disinfectant level allowed and 2 mg/L represents a common operational target.^51–53^ Additionally, the World Health Organization considers chlorine concentrations up to 5 mg/L safe for short-term exposure, while suggesting that residuals of 0.2–0.5 mg/L are generally sufficient to maintain microbial control at the point of delivery.^52^ While concentrations of 2 and 4 mg/L chlorine are typically achieved at the treatment plant, they are uncommon in domestic HWNs due to disinfectant decay during distribution. Nonetheless, they are realistic for point-of-entry or in-line chlorination systems and align with widely accepted thresholds for safe consumption.

The concentrations of 4, 8, and 20 mg/L were selected for assessing SSHP decay. The lowest concentration (4 mg/L) was included for direct comparison with free chlorine at the same level. This concentration also reflects average SSHP residuals (4–5 mg/L) measured at distal points in two full-scale drinking water distribution systems treated with an initial 8 mg/L SSHP dose.^39^ The 8 mg/L target was selected based on NSF 60 certification, which permits continuous use of SSHP at this residual concentration in drinking water.^39,46^ However, preliminary testing showed that 8 mg/L SSHP decayed rapidly in chloraminated tap water from one of the two sampled test sites. To maintain measurable oxidant residuals in this chemically complex matrix, a higher dose of 20 mg/L was used in subsequent experiments. This concentration aligns with field applications where SSHP or hydrogen peroxide has been applied at >25 mg/L during commissioning, remediation, or to overcome elevated system demand.^39,40,54^ The concentrations selected in this study were intended to reflect realistic operational constraints and application contexts, rather than to necessarily provide an equal-dose or contact-time (CT) based comparison of intrinsic disinfectant potency, as such comparisons may not capture disinfectant performance under conditions of rapid, matrix-dependent decay characteristic of hot water systems.

#### 2.3.2 Hot Water Decay Protocol

Disinfectant stability was assessed in (i) Milli-Q ultrapure water, (ii) SYTW without organics, (iii) SYTW with either tannic or humic acid added as the sole organic source, and (iv) municipal tap water (City of Toronto, Toronto, ON, Canada). When organic matter was added to SYTW it was either added (i) prior to the addition of the disinfectants to allow for breakpoint correction or (ii) following the addition of the disinfectants to replicate a “contamination” event. Municipal tap water was obtained from a large university campus building built in the 1960s, as well as a major shopping center, both located downtown Toronto. The City of Toronto employs chlorination for primary disinfection and chloramination for residual disinfection; therefore, both free and total chlorine were measured at each time point during tap water assays.

Stock disinfectant solutions were prepared in demand-treated glassware. Following the addition of the disinfectant, the stock solutions were pH-adjusted (pH 7.2–7.6; except for those prepared in tap water which were not adjusted) and, if necessary, breakpoint corrected for immediate demand. This pH range was selected to reflect the native pH of Toronto tap water^50^ and to maintain consistent, representative conditions across treatments, rather than to optimize performance for any specific disinfectant. In the case of the simulated contamination events, the organic carbon source was added to the stock solution following pH- and concentration-adjustment. Once the desired final disinfectant concentration was reached, the stock solution was dispensed into three demand-treated 25 mL glass-stoppered Erlenmeyer flasks. The outside of these flasks was covered in tinfoil to prevent light exposure. At the beginning of each experiment, the 25 mL flasks were filled to overflowing before then being stoppered to ensure there was no headspace. The stoppered flask was then sealed with polytetrafluoroethylene thread tape, a nitrile sleeve was pulled tightly over flask, and the nitrile sleeve was tied off to prevent liquid loss. Once sealed, the 25 mL flasks were completely submerged in a covered hot water bath set to 55 or 60 °C. Several control trials using empty sealed vessels confirmed that no water ingress occurred during the multiday submersion. The temperature in the hot water bath was continuously monitored by (i) the hot water bath thermocouple, (ii) a fully submerged glass thermometer, and (iii) a digital thermometer with a fully submerged thermocouple.

Following 24, 48, or 72 h of incubation, one of the three replicate 25 mL flasks was removed from the hot water bath and placed on ice to cool. Once cooled, the flasks were sampled for disinfectant concentration.

An exponential decay model of the form *C­(t) = C*_0_ *×* e^*–kt*^ was fit to the total chlorine and SSHP data sets from municipal tap water using nonlinear least-squares regression, where *C­(t)* is the concentration at time *t*, *C*_0_ is the initial concentration, and *k* is the decay rate constant. The fitted curves were used to estimate the half-lives of the disinfectants (*t*_*1/2*_ *= ln(2)/k*) and assess model fit using the coefficient of determination (*R* ^2^). Separate models were fit for each disinfectant and water source. Free chlorine data were not modeled due to rapid depletion and the absence of clear exponential decay behavior.

### 2.4 Kill-Time Assays: Assessing the Impact of Prolonged Incubation at High Temperature on SSHP Efficacy

SYTW with humic acid was breakpoint treated to reach either 8 mg/L of SSHP or 2 mg/L of free chlorine and incubated for 24–72 h at 55 or 60 °C. Following incubation, the solutions were placed in a refrigerator to halt any further heat-driven decay. *L. pneumophila* was washed twice in fresh, filter-sterilized SYTW with humic acid and standardized to a cell density of 1 × 10^7^ CFU/mL using OD_600_ standardization as described in Section 2.2. The previously heated solutions were removed from the fridge, heated back to 45 °C, and an aliquot of the standardized *L. pneumophila* was added to reach a final cell density of 1 × 10^5^ CFU/mL. In a HWN, as hot water leaves the heating source it will cool both from mixing with cold water and from heat loss. As such, 45 °C was used to simulate expected temperatures as water leaves the hot water source and travels downstream to *L. pneumophila*-contaminated premise plumbing.

At 5 min intervals, an aliquot was removed from the culture and neutralized by dilution in filter-sterilized, 100 mM phosphate buffer containing neutralizing compounds. SSHP was neutralized using 1 g/L protease peptone and 0.667 g/L of catalase from bovine liver. Once the dilutions were vortexed and allowed to sit for a period of 2–3 min, filter-sterilized sodium thioglycolate was added to achieve a final concentration of 1 g/L for silver complexation. Free chlorine was neutralized using a solution of 0.1 M potassium thiosulfate. Serial dilutions were plated in triplicate on BCYE-α plates. Plates with between 30 and 300 colonies were counted to give an estimation of viable cell concentration in CFU/mL. Detection limits were assay-specific, calculated from the actual starting cell concentrations and a minimum quantifiable threshold of 30 CFU per plate. This same protocol was also conducted using freshly prepared solutions of 8 mg/L SSHP and 2 mg/L of free chlorine to determine if the prolonged incubation for 24–72 h at 55 and 60 °C impacted the biocidal efficiency of the disinfectant.

Following the municipal tap water decay assays, an additional kill-time experiment was performed to evaluate whether the SSHP residual expected after 72 h of incubation at 60 °C would retain bactericidal activity. In the two tap water sources tested under these conditions, 20 mg/L SSHP decayed to mean residual concentrations of 2.7 and 5.2 mg/L, respectively; therefore, a representative residual concentration of 4 mg/L was selected (Figure 5b; Section 3.2.3). Fresh 4 mg/L SSHP solutions were prepared in SYTW supplemented with humic acid and equilibrated to 45 °C. *L. pneumophila* cultures were washed twice in SYTW with humic acid, adjusted to 1 × 10^7^ CFU/mL, and added to the disinfectant solution to reach 5 × 10^4^ CFU/mL. Sampling, neutralization, dilution, plating, incubation, and log_10_ reduction calculations were performed as described in Section 2.2.

Disinfectant performance was assessed by comparing log_10_ reduction values (i.e., log removal) of *L. pneumophila* across treatments at each time point. Pairwise comparisons were conducted using Welch’s two-tailed *t* tests in Microsoft Excel. Comparisons were made between freshly prepared and thermally aged disinfectant solutions, as well as against untreated controls. A *p*-value <0.05 was considered statistically significant.

## 3 Results and Discussion

### 3.1 Effect of Temperature on SSHP Efficacy

To decouple intrinsic oxidant performance from the complex chemistry of municipal water, SSHP was evaluated in a defined synthetic tap water (SYTW). At both 35 and 45 °C in SYTW, *L. pneumophila* viability was not impacted by temperature alone, even after 2 h of incubation (Figure S1). In contrast, incubation at 55 °C resulted in a 1.74-log reduction within 30 min, indicating that this temperature is inherently biocidal (Figure 1). However, the addition of SSHP substantially accelerated bacterial inactivation at all temperatures, and increasing temperature consistently enhanced SSHP disinfection performance.

**Figure 1:**
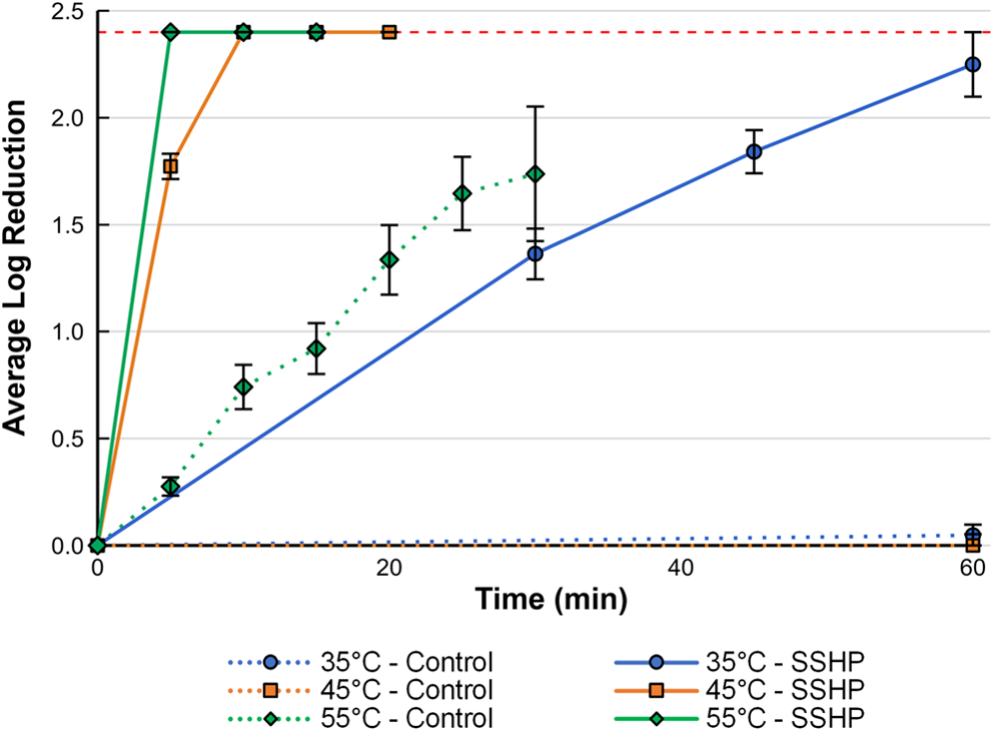
The effect of temperature on silver-stabilized hydrogen peroxide’s (SSHP) biocidal efficiency when employed against 1 × 10^5^ colony forming units per milliliter of *L. pneumophila* at 35 °C, 45 °C, and 55 °C. The dotted lines represent *L. pneumophila* log reductions in the control conditions for each temperature (i.e., synthetic tap water without disinfectant). The solid lines represent *L. pneumophila* log reductions in the 20 mg/L SSHP conditions for each temperature. The red dashed line represents the limit of detection. Data are presented as mean ± standard error of the mean (*n* = 3–6).

At 35 °C, 20 mg/L SSHP required more than 45 min to achieve a 2-log reduction, whereas at 45 °C the same dose reduced cell counts below the detection limit (>2.4-log) in under 10 min (Figure 1). At 55 °C, 20 mg/L SSHP achieved >2.4-log reduction within just 5 min, while heat alone produced only a 0.28-log reduction over the same interval. These results indicate that increasing temperature markedly accelerated SSHP-mediated inactivation, exceeding the reduction achieved by heat alone over the same time.

Together, these results indicate that SSHP efficacy increased with temperature across a range relevant to warm and hot water systems. This temperature-dependent behavior is consistent with prior reports of enhanced SSHP efficacy at elevated temperatures.^26,29,31^ Because these assays used intentionally high bacterial densities, the observed kill-time performance also likely represents a conservative estimate of efficiency relative to typical HWN planktonic concentrations.

### 3.2 Oxidant Decay in Hot Water

#### 3.2.1 Decay at 55 °C—Milli-Q and SYTW without Organics

When incubated at 55 °C for 72 h in Milli-Q ultrapure water, free chlorine and SSHP decayed at markedly different rates (Figure S2). On average, 4 mg/L of free chlorine declined by 49% over the three-day period, whereas the hydrogen peroxide component of SSHP remained relatively stable, exhibiting less than 2% loss after 72 h.

In contrast, when incubated at 55 °C for 72 h in organic-free SYTW, both disinfectants decayed at comparable rates across the tested concentration ranges (Figure 2). By the end of the incubation period, free chlorine declined by an average of 27% and 33% in the 2 mg/L and 4 mg/L conditions, respectively. Similarly, hydrogen peroxide in the SSHP treatments decreased by 35% and 24% in the 4 mg/L and 8 mg/L conditions, respectively.

**Figure 2:**
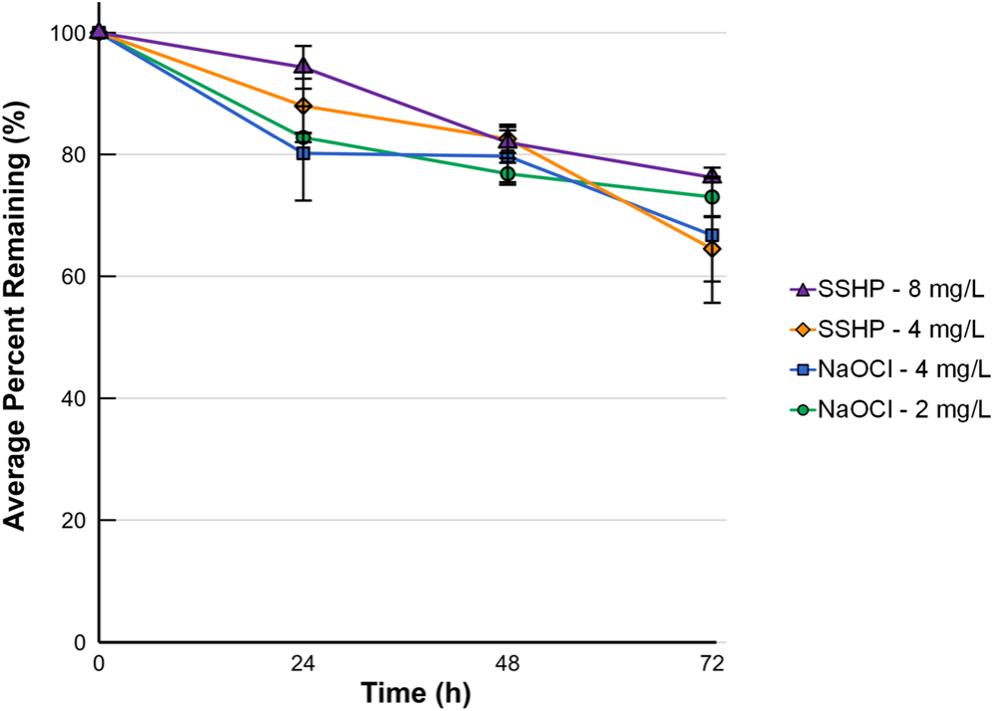
The average remaining percentage of silver-stabilized hydrogen peroxide (SSHP) and sodium hypochlorite (NaOCl; i.e., chlorine) following 72 h of incubation at 55 °C in synthetic tap water without organics. Free chlorine was measured for NaOCl, and hydrogen peroxide was measured for SSHP. Data are presented as mean ± standard error of the mean (*n* = 3–6).

The increased SSHP decay observed in SYTW relative to Milli-Q indicates that inorganic constituents alone can impose measurable demand on hydrogen peroxide at elevated temperature, narrowing the apparent stability difference between SSHP and free chlorine. Based on the composition of SYTW (Table S2), this behavior is most plausibly driven by trace redox-active metals, particularly iron and copper, which catalyze hydrogen peroxide decomposition through Fenton and Fenton-like reactions, resulting in direct oxidant loss via redox cycling and reactive intermediate formation.^55^ Further, while carbonate species do not directly contribute to hydrogen peroxide decomposition, their presence in SYTW may influence the fate of reactive intermediates generated during metal-catalyzed decay by scavenging hydroxyl radicals, thereby altering downstream reaction pathways.^56,57^ Together, these results demonstrate that inorganic constituents alone can drive hydrogen peroxide decay in simplified but nonideal matrices. In HWNs, inorganic compounds may leach from corroding infrastructure or precipitate during heating, particularly in systems with extended stagnation or storage tanks.^58^ Our results in SYTW, highlight the potential for inorganic constituents to contribute meaningfully to SSHP demand in real water systems.

#### 3.2.2 Decay at 55 °C—SYTW with Organics

##### 3.2.2.1 Breakpoint Treated

When SYTW was prepared with organic matter and breakpoint treated to satisfy initial oxidant demand prior to disinfectant addition, free chlorine decayed rapidly at 55 °C (Figure 3). In the presence of tannic acid, 2 mg/L free chlorine was completely depleted within 24 h. At 4 mg/L, free chlorine concentrations declined by an average of 77% at 24 h, 89% at 48 h, and 97% at 72 h. In contrast, SSHP hydrogen peroxide exhibited greater stability in the presence of organics than in organic-free SYTW, and no breakpoint correction was required. After 72 h of incubation with tannic acid, only 9.3% and 7.4% of the initial hydrogen peroxide concentrations were lost in the 4 mg/L and 8 mg/L SSHP treatments, respectively.

**Figure 3:**
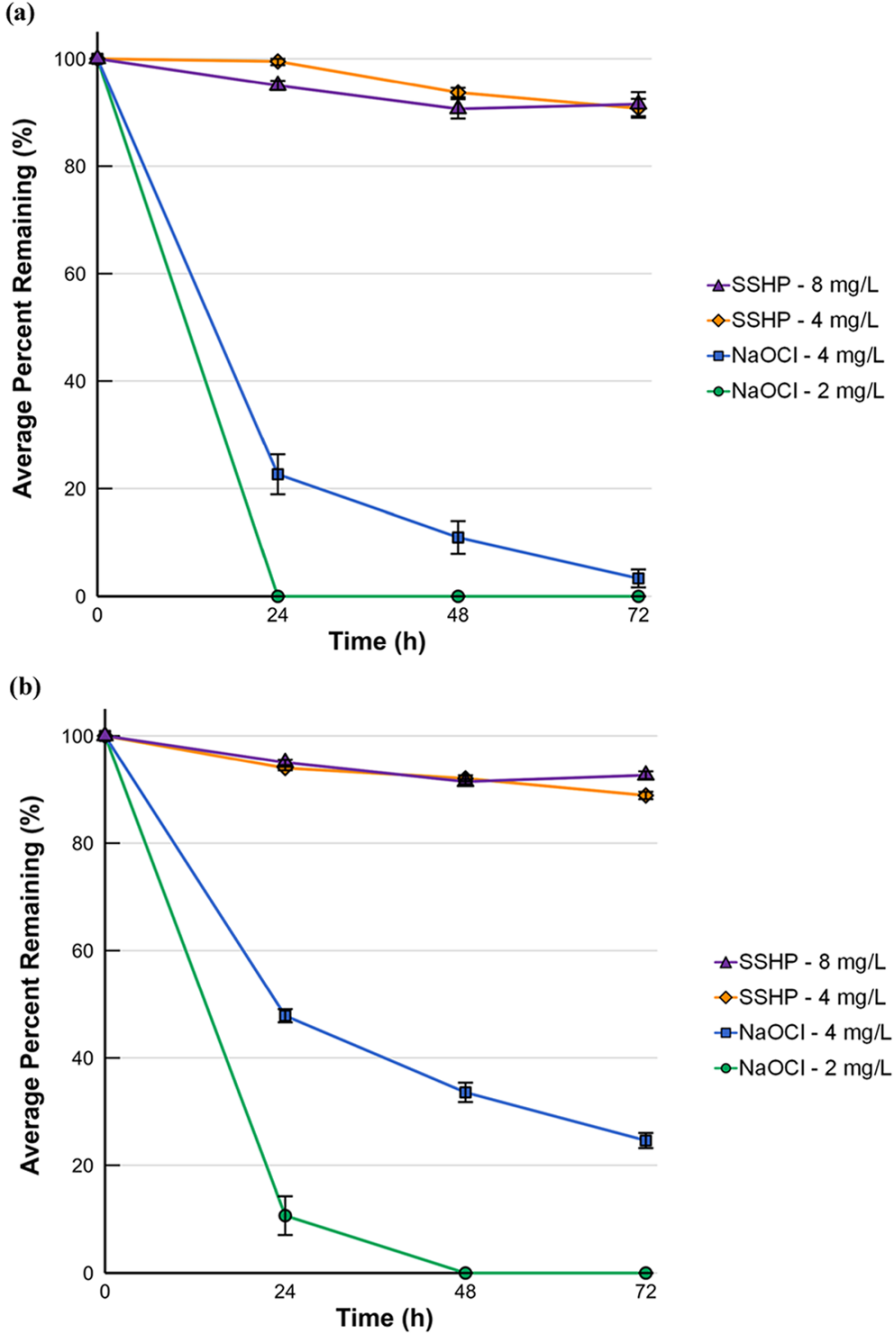
The average remaining percentage of silver-stabilized hydrogen peroxide (SSHP) and sodium hypochlorite (NaOCl; i.e., chlorine) following 72 h of incubation at 55 °C in synthetic tap water containing organics that had been breakpoint treated. The organics used were (a) tannic and (b) humic acid, added to achieve a total organic carbon concentration of 1.4 mg/L. Free chlorine was measured for NaOCl, and hydrogen peroxide was measured for SSHP. Data are presented as mean ± standard error of the mean (*n* = 3–10).

Similar trends were observed with SYTW containing humic acid. At 2 mg/L, free chlorine declined by 89% within 24 h and fell below the detection threshold (0.05 mg/L) by 48 h. In the 4 mg/L condition, free chlorine decayed by 52% at 24 h, 66% at 48 h, and 75% at 72 h. In comparison, SSHP hydrogen peroxide concentrations decreased by only 9.3% and 11.1% in the 4 mg/L and 8 mg/L SSHP treatments, respectively, after 72 h. These results indicate that SSHP maintained greater stability than free chlorine in the presence of organic matter, even when immediate chlorine demand was satisfied by breakpoint treatment, suggesting that its persistence may be less sensitive to changes in organic load. This property may be advantageous in scenarios where organic contamination is introduced downstream of SSHP treatment and is consistent with reports of effective SSHP application in high-organic water systems.^39^

##### 3.2.2.2 Simulated Contamination

When organic matter was introduced following breakpoint disinfection, simulating a contamination event in previously organic-free SYTW, free chlorine decayed most rapidly (Figure S3). In the presence of tannic acid, both 2 mg/L and 4 mg/L free chlorine concentrations dropped below the detection threshold within 24 h. Under the same conditions, SSHP exhibited much greater stability, with 4 mg/L and 8 mg/L SSHP treatments showing average hydrogen peroxide losses of only 13% and 14%, respectively, after 72 h.

When humic acid was added as a contaminant, free chlorine at 2 mg/L was completely depleted within 24 h. In the 4 mg/L condition, free chlorine declined by 72% at 24 h, 89% at 48 h, and 96.5% at 72 h. In contrast, SSHP hydrogen peroxide concentrations declined by only 12% and 11.9% in the 4 mg/L and 8 mg/L SSHP treatments, respectively, over 72 h. Together, these results demonstrate that SSHP retains greater stability than free chlorine during organic contamination events under controlled synthetic conditions.

SSHP degraded fastest in SYTW containing only inorganic constituents but was more stable when organics were also present. While initially counterintuitive, this behavior is consistent with interactions between organic compounds and reactive inorganics in SYTW, particularly transition metals, that would otherwise likely catalyze SSHP decomposition.^55^ NOM can bind dissolved and particulate metals through functional groups, such as carboxyl and hydroxyl moieties, reducing the availability of redox-active species in solution.^59^ In this context, organic matter likely stabilized SSHP indirectly by limiting the catalytic activity of these species and suppressing hydrogen peroxide decomposition. These findings suggest that SSHP stability is governed by the balance between redox-active inorganics and complexing organic constituents, with organic matter potentially mitigating inorganics-driven oxidant decay under realistic conditions. In contrast, chlorine appears to be more reactive toward organic matter specifically, which likely contributes to its more rapid decay under comparable conditions.

#### 3.2.3 Decay at Elevated Temperature in Municipal Tap Water

Although SYTW enabled controlled comparisons, it does not capture all sources of oxidant demand present in drinking water matrices. When incubated at 60 °C in SYTW amended with humic acid, 2 mg/L free chlorine decayed completely within 24 h, whereas 8 mg/L SSHP exhibited greater thermal stability, decreasing by approximately 4% within 48 h and 14.2% within 72 h (Figure S4). These results suggest that SSHP stability is maintained under elevated temperature conditions even in the presence of organic matter, motivating further testing in more complex municipal water matrices. Chlorine and SSHP were therefore subsequently incubated in municipal tap water collected from two downtown Toronto sites.

Fresh tap water samples from the university building and shopping center contained average starting total chlorine concentrations of 1.76 mg/L and 1.89 mg/L, and free chlorine concentrations of 0.35 mg/L and 0.18 mg/L, respectively (*n* = 3). In untreated university water heated to 60 °C, total chlorine declined by 55%, 78%, and 90% after 24, 48, and 72 h of incubation (final residual = 0.17 mg/L; Figure 4). Free chlorine in those same samples declined by 62% within 24 h and was no longer detectable after 48 h. In the shopping center water, total chlorine declined by 58%, 76%, and 86% at 24, 48, and 72 h, respectively (final residual = 0.27 mg/L). Free chlorine in the shopping center water fell to 0.08 mg/L at 24 h, remained detectable at 48 h (0.07 mg/L), and dropped below detection by 72 h. Exponential decay models fit to the total chlorine data revealed similar decay rate constants in both water types (*k* = 0.032–0.035 h^–1^), corresponding to half-lives of 19.7 h (university) and 21.5 h (shopping center). In contrast, free chlorine in both samples decayed too rapidly to support exponential modeling.

**Figure 4:**
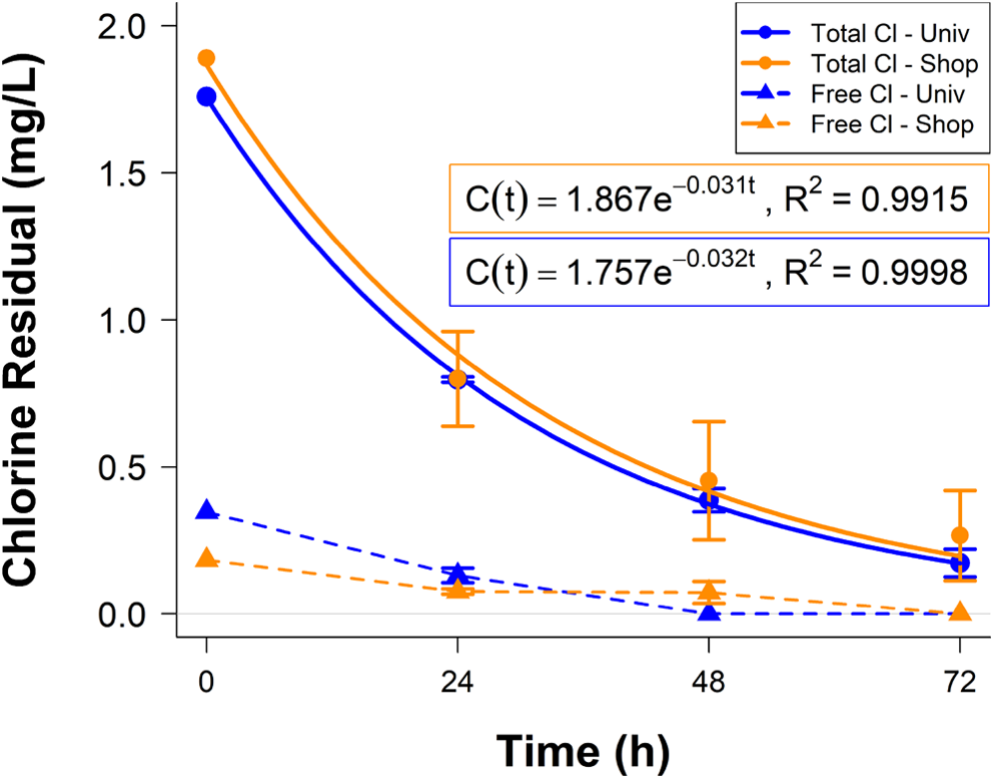
Total and free chlorine concentrations in untreated municipal tap water from two City of Toronto sources incubated at 60 °C for 72 h. The two sources were a large university building (“Univ”; blue) and a major shopping center (“Shop”; orange), both located in downtown Toronto. Solid lines represent exponential decay of total chlorine, modeled using the equation *C­(t) = C*_*0*_ *×* e^*–kt*^, where *C­(t)* is the concentration at time *t*, *C*_*0*_ is the initial concentration, and *k* is the decay rate constant. Nonlinear least-squares regression was used to fit the model. Dashed lines represent free chlorine concentrations, which decayed too rapidly to support exponential modeling. Data are presented as mean ± standard error of the mean (*n* = 3).

When chloraminated municipal water was supplemented with chlorine or SSHP and incubated at 60 °C, SSHP showed markedly improved persistence relative to chlorine (Figure 5). Notably, these waters were chemically naïve to hydrogen peroxide and contained pre-existing chlorine/chloramine residuals, conditions that are expected to accelerate hydrogen peroxide decay by increasing its demand rather than favoring its persistence.^60,61^ In the university water, 4 mg/L free chlorine declined below detection within 24 h (Figure 5a), with total chlorine falling to 0.15 mg/L in 24 h and becoming undetectable by 48 h. In contrast, the same chlorine dose in the shopping center water retained 0.35 mg/L free chlorine and 0.45 mg/L total chlorine at 24 h, but both residuals decayed completely by 48 h.

**Figure 5:**
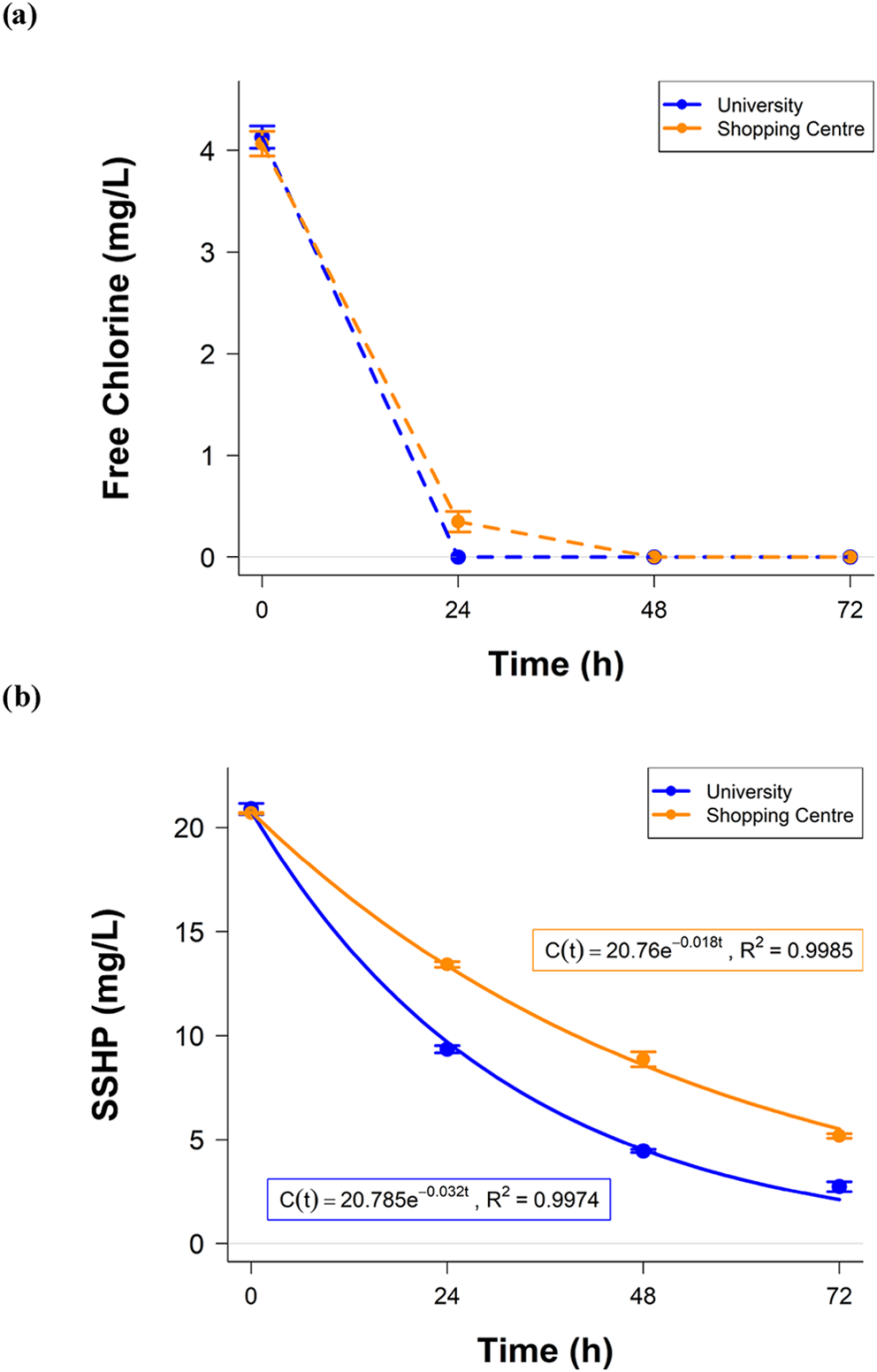
The average remaining concentrations of (a) free chlorine and (b) silver-stabilized hydrogen peroxide (SSHP) following 72 h of incubation at 60 °C in City of Toronto municipal tap water from two sources. The two sources were a large university building (“University”; blue) and a major shopping center (“Shopping Centre”; orange), both located in downtown Toronto. Solid lines represent exponential decay of SSHP, modeled using the equation *C­(t) = C*_*0*_ *×* e^*–kt*^, where *C­(t)* is the concentration at time *t*, *C*_*0*_ is the initial concentration, and *k* is the decay rate constant. Nonlinear least-squares regression was used to fit the model. Dashed lines represent free chlorine concentrations, which decayed too rapidly to support exponential modeling. In both sources, the initial free chlorine and SSHP concentrations were approximately 4 and 20 mg/L, respectively. Data are presented as mean ± standard error of the mean (*n* = 3).

SSHP decay followed an exponential trajectory, with decay rates differing between the two water sources (Figure 5b). In the university water, SSHP concentrations declined by 55%, 79%, and 87% after 24, 48, and 72 h, respectively (final residual = 2.73 mg/L). In the shopping center water, SSHP exhibited greater stability, declining by only 35%, 57%, and 75% over the same period (final residual = 5.18 mg/L). Exponential modeling yielded SSHP decay constants of 0.030 h^–1^ (university) and 0.018 h^–1^ (shopping center), corresponding to half-lives of 23.0 and 39.1 h, respectively.

In both waters, SSHP therefore exhibited longer half-lives than the total and free chlorine residuals, despite being applied to chloraminated water from systems with a long-standing history of chlorine and chloramine treatment. Such systems would be expected to have partially depleted chlorine-reactive constituents due to prolonged exposure to chlorine-based residuals and primary disinfection at high doses, which would be expected to favor chlorine persistence in these tests. In contrast, hydrogen peroxide–reactive species, including residual chlorine and chloramine,^60,61^ remain present, yet SSHP still exhibited greater persistence under these conditions.

### 3.3 The Effect of Thermal Aging on Disinfectant Efficiency

#### 3.3.1 Retention of Disinfectant Efficacy Following Thermal Aging

At 45 °C, SYTW amended with tannic acid inhibited *L. pneumophila* in the absence of disinfectant (Figure S5), indicating that the organic matrix itself influenced bacterial viability. To avoid this confounding effect, subsequent kill-time assays were conducted exclusively in SYTW amended with humic acid, which did not exhibit independent antimicrobial activity.

After 72 h of incubation in SYTW with humic acid at 55 °C, SSHP fully retained its bactericidal efficacy, consistent with its measured chemical stability in decay assays (*p* > 0.05; Figure 6). In contrast, chlorine efficacy declined substantially following the same incubation period, mirroring its rapid decay (Figure S6). While freshly prepared 2 mg/L free chlorine solutions reduced *L. pneumophila* to below the detection limit within 5 min, chlorine solutions aged for 72 h in SYTW achieved an average log reduction of only 0.06 after 30 min at 45 °C and did not perform significantly better than the no-disinfectant control (*p* > 0.05).

**Figure 6:**
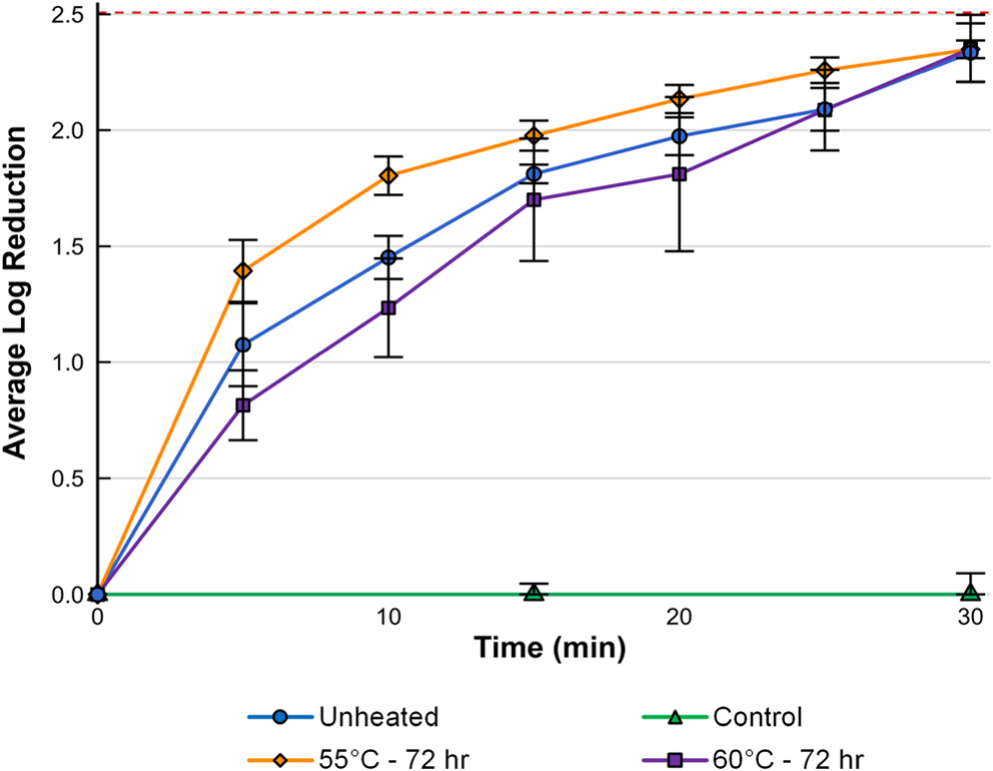
Effect of thermal aging on the biocidal activity of silver-stabilized hydrogen peroxide (SSHP; 8 mg/L) in synthetic tap water supplemented with humic acid (1.2–1.4 mg/L total organic carbon). To simulate prolonged storage in hot water tanks, SSHP solutions were incubated at 55 or 60 °C for 72 h. Disinfection assays were then performed at 45 °C against *L. pneumophila* (initial concentration: 1 × 10^5^ colony forming units per milliliter), representing temperatures typical of distal outlets in hot water systems. All SSHP treatments exhibited sustained biocidal activity, with thermally aged solutions showing no significant loss of efficacy compared to freshly prepared SSHP (*p*-value >0.05). In contrast, the untreated control showed no inactivation over the 30 min period. The red dashed line indicates the limit of detection. Data are presented as mean ± standard error of the mean (*n* = 3–4).

At 60 °C, rapid chlorine decay constrained kill-time assays to solutions aged for 24 h, whereas SSHP retained sufficient residual to permit evaluation following longer incubation. Solutions containing 8 mg/L SSHP retained full bactericidal efficacy after 72 h of thermal aging at 60 °C in SYTW, showing no significant difference from freshly prepared solutions (*p* > 0.05; Figure 6). Conversely, after only 24 h of thermal aging, 2 mg/L free chlorine solutions exhibited significantly reduced efficacy (*p* = 2.15 × 10^–4^), achieving only 0.05-log removal of *L. pneumophila* after 30 min at 45 °C (Figure S6).

These results indicate that SSHP retains antimicrobial efficacy following prolonged thermal exposure, whereas chlorine exhibited reduced effectiveness under the same conditions, consistent with its observed decay and underscoring the importance of disinfectant stability for sustained control in HWNs.

#### 3.3.2 Efficacy of Representative SSHP Residuals after Thermal Aging

As decay assays showed that 20 mg/L SSHP declined to 2.7 mg/L and 5.2 mg/L in municipal tap waters after 72 h of incubation at 60 °C, a concentration of 4 mg/L SSHP was selected as a representative residual for subsequent kill-time assays in SYTW amended with humic acid. This concentration reflects the range of SSHP residuals measured under network-relevant thermal conditions following decay, while the use of SYTW allowed efficacy to be evaluated under controlled matrix conditions.

When tested at 45 °C, 4 mg/L SSHP achieved greater than 1-log_10_ reduction of *L. pneumophila* within 10 min and exceeded 2-log_10_ reduction within 25 min (Figure S7). These results demonstrate that SSHP residuals persisting after prolonged thermal exposure in municipal water remained bactericidal over short contact times, linking chemical persistence to retained antimicrobial efficacy. This suggests that such residuals may continue to exert antimicrobial activity during periods of storage or stagnation in HWNs, where extended residence times can influence microbial risk.

### 3.4 Implications for Hot Water Network Disinfection

#### 3.4.1 System-Level Constraints on Disinfectant Performance in Hot Water Networks

Thermal treatment remains a primary control strategy in hot water networks (HWNs), but its implementation is energetically costly and operationally constrained. Even modest reductions in hot water set points (5–6 °C) could yield substantial residential energy savings (e.g., up to $350 million annually in Canada; Table S1). Further, in practice, water temperatures decrease as water moves away from heating sources and through distal sections of HWNs, frequently entering ranges that no longer suppress microbial growth and may instead permit pathogen persistence or proliferation. As a result, thermal control alone is often spatially/temporally inconsistent across building-scale networks.

HWNs are therefore commonly managed using a combination of thermal control and chlorine-based disinfection. However, neither approach has proven consistently effective for controlling opportunistic premise plumbing pathogens, such as *L. pneumophila*.^34,36^ Repeated thermal exposure has been associated with the emergence of thermotolerant *Legionella* strains,^35^ while chlorine residuals are known to decay rapidly under the elevated temperatures and extended residence times characteristic of HWNs. Under stagnant hot water conditions, increased reaction kinetics and sustained oxidant demand accelerate chlorine decay, limiting the persistence of disinfectant residuals precisely where extended disinfection protection is most needed.

Together, these constraints highlight that HWNs impose chemical, thermal, and hydraulic conditions that differ fundamentally from those of cold water distribution systems. Disinfectants that perform adequately under cold water conditions may therefore fail to maintain residual activity in HWNs, not due to insufficient intrinsic antimicrobial potency, but because their chemical stability is incompatible with prolonged exposure to heat and stagnation. This distinction underscores the importance of evaluating disinfectants against system-relevant stressors, rather than relying solely on short-term efficacy measurements conducted under controlled or idealized conditions.

#### 3.4.2 Residual Persistence as a Prerequisite for Sustainable HWN Disinfection

The results of this study indicate that disinfectant performance in HWNs is governed less by short-term antimicrobial potency than by the ability to retain a residual under prolonged exposure to heat, stagnation, and oxidant demand. Under such conditions, performance metrics based solely on concentration–time relationships (i.e., CT-based metrics) may not fully capture disinfectant effectiveness. In this study, free and total chlorine frequently declined below internationally recognized minimum thresholds for residual protection (≥0.2 mg/L free chlorine and ≥0.5 mg/L total chlorine)^51,52,62^ within timeframes that HWNs often remain stagnant. In several cases, free chlorine fell below 0.2 mg/L in less than 24 h, while total chlorine declined below 0.5 mg/L within approximately 40 h, despite starting from disinfectant residuals commonly regarded as effective for microbial control in distribution systems and that exceeded typical operational targets.

These observations are consistent with well-established, temperature-dependent chlorine decay kinetics and with prior reports linking hot water conditions to accelerated disinfectant loss and increased disinfection byproduct formation.^21–23^ Under such conditions, maintaining protective chlorine residuals would likely require frequent redosing or elevated initial concentrations, both of which present operational, chemical, and regulatory challenges. Notably, the rapid chlorine decay observed here occurred in municipal waters with a long-standing history of chloramine use, under which readily chlorine-reactive species would typically be expected to be depleted. This rapid decay indicates that substantial oxidant demand can persist under HWN-relevant thermal conditions in chlorinated/chloraminated systems.

In contrast, SSHP retained multiday residuals under comparable thermal and hydraulic conditions and remained bactericidal over short contact times even after prolonged incubation, linking chemical persistence directly to retained antimicrobial efficacy. Importantly, this persistence was observed in municipal waters containing residual chlorine and chloramine, and with no prior conditioning to hydrogen peroxide. These conditions are expected to increase hydrogen peroxide demand, both through temperature-accelerated reactions with chloramine species and hypochlorite^60,61^ and with peroxide-reactive compounds not normally consumed by chlorine/chloramine. Field studies similarly report effective hydrogen peroxide/SSHP persistence in systems where elevated temperature, long residence times, or strong demand limit chlorine’s performance.^39,40,54^

Disinfectants capable of retaining residual activity after extended exposure to heat and stagnation could, in theory, enable HWNs to operate safely at moderately lower temperature set points, thereby reducing energy demand without compromising public health protection. The results of this study illustrate that, by evaluating disinfectants against system-specific constraints, it becomes possible to identify candidates whose properties align with the hydraulic, thermal, and chemical conditions of the system in which they must operate. When evaluated against the stagnation, elevated temperature, and persistent oxidant demand characteristic of HWNs, SSHP met the criteria considered necessary for effective HWN disinfection. In other systems, however, different disinfectants—or indeed combinations of disinfectants—may be more suitable.

At the same time, bulk-water residual persistence alone does not guarantee effective pathogen control in operational HWNs. Real systems contain biofilms, hydraulic dead zones, inorganic/organic deposits, and spatial temperature gradients that reduce oxidant exposure, increase demand, or create refuges for microbial survival. These constraints are further influenced by factors such as pipe material composition (e.g., copper, lead, cement, and plastics) and pH, which can alter surface reactivity and the availability of redox-active species, thereby affecting hydrogen peroxide stability. Some protozoan hosts such as *Acanthamoeba* spp. can also internalize bacteria like *L. pneumophila* and protect those intracellular pathogens from disinfection, allowing persistence even under aggressive treatment regimes.^63–66^ Such interactions have been demonstrated in hot water microcosms, where neither chlorine nor monochloramine consistently eliminated *L. pneumophila*, with survival attributed to host protection and biofilm reservoirs.^6^ Accordingly, residual persistence can be considered a necessary but not sufficient condition for pathogen control in HWNs, and future work should evaluate realized disinfectant performance using longer-term, pilot-scale studies that incorporate realistic hydraulic, thermal, and biological complexity.

Taken together, the approach applied here—identifying system-specific stressors, quantifying disinfectant stability under those conditions, and linking residual persistence to retained antimicrobial efficacy—provides a generalizable framework for evaluating disinfectants in challenging water systems. Although SSHP and HWNs are used as case examples here, the broader implication is that effective and sustainable pathogen control depends on aligning disinfectant physicochemical properties with the thermal, hydraulic, and chemical constraints of the system in which they must operate. Applying this framework may support the development of more diversified, resilient, and sustainable disinfection strategies across a range of engineered water environments, from building-scale plumbing to larger distribution networks.

## 4 Conclusions

Under HWN-relevant conditions, chlorine residuals frequently fell below protective thresholds, whereas SSHP maintained multiday residuals. SSHP retained antimicrobial activity after prolonged thermal exposure in both synthetic and municipal waters, and its bactericidal activity against *L. pneumophila* increased with temperature. Together, these findings demonstrate the importance of a systems-level approach that aligns disinfectant properties with the thermal and chemical constraints of the target system, informing more resilient and sustainable water-management strategies.

## Supplementary Material



## Data Availability

The data supporting the findings of this study are available from the corresponding author upon request.

## Notes

Tables summarizing hot water energy-use estimates and synthetic drinking water composition, and figures reporting disinfectant decay kinetics, temperature- and organic matter-dependent *L. pneumophila* viability, and SSHP biocidal activity under hot-water-relevant conditions (PDF)
