# Supplementary material for: Alternative Disinfection in Hot Water Networks: Persistence and Antimicrobial Efficacy of Silver-Stabilized Hydrogen Peroxide for Legionella pneumophila Control
Source: ACS ES T Water. 2026 May 23;6(6):3736–46. doi: 10.1021/acsestwater.6c00121 (PMC13271086; doi:10.1021/acsestwater.6c00121)
Supplement: Supplementary file 1 [file ew6c00121_si_001.pdf]

Alternative Disinfection in Hot Water Networks: Persistence and Antimicrobial Efficacy of Silver-Stabilized Hydrogen Peroxide for *Legionella pneumophila* Control

Nate Clark[a], Lynda H. McCarthy[a], & Steven N. Liss[a,b,c*]

[a] Toronto Metropolitan University, Toronto, Ontario, M5B 2K3, Canada

[b] Queen's University, Kingston, Ontario, K7L 3N6, Canada

[c] Stellenbosch University, Stellenbosch, Western Cape, 7602, South Africa

*Corresponding author

Email: steven.liss@torontomu.ca

Table S1. Estimated hot water energy use and cost savings from 5.6°C (10°F) setpoint reduction in Canadian households. Estimates are based on national average electricity prices and assume, for illustrative purposes and ease of analysis, that all Canadian households use electric water heating. Actual energy savings will vary by energy source, household type, and region. Percent savings are based on estimates from the United States Department of Energy, which suggest that a 10°F (≈5.6°C) reduction in water heater setpoint yields annual hot water energy savings of 3 – 5%.

Variable	Amount
Total Canadian households[1]	16,284,235
Total household energy use[2]	361,111,110,000 kWh
Total hot water energy use[3]	62,111,110,920 kWh
Total electricity cost[4]	$6.99 billion
10°F decrease – 3% saving[5]	$209.6 million/year
10°F decrease – 5% saving[5]	$349.4 million/year

[1] Statistics Canada, 2021 Census. https://www12.statcan.gc.ca/census-recensement/index-eng.cfm.
Represents the number of private households in Canada.

[2] Statistics Canada. https://www150.statcan.gc.ca/n1/daily-quotidien/240319/dq240319d-eng.htm.
Annual residential electricity consumption across all Canadian households.

[3] Natural Resources Canada. https://natural-resources.canada.ca/energy-efficiency/products/water-heaters/13735.
Estimated that ~17% of total household energy use goes to hot water heating.

[4] Electricity Canada. https://www.electricity.ca/knowledge-centre/the-grid/customer/electricity-rates/.
Based on an average national electricity rate of ~$0.112/kWh.

[5] U.S. Department of Energy. https://www.energy.gov/energysaver/articles/15-ways-save-your-water-heating-bill.
Estimated that lowering water heater temperature by 10°F can save 3–5% of hot water energy costs annually

Table S2. Inorganic/organic parameters reported in the 2021 Toronto Drinking Water Analysis (City of Toronto, 2021) and in the synthetic drinking water medium used in this study.

Inorganic/organic parameter	2021 Toronto Drinking Water Analysis average concentration (mg/L) or value	Synthetic Toronto drinking water concentration (mg/L) or value	Synthetic Toronto drinking water inorganic source(s)
Na	14	15.1	NaF, $B_4Na_2O_7$, $NaNO_3$, $Na_2MoO_4 \cdot 2H_2O$, $NaHCO_3$
Fe	0.03	0.03	$FeSO_4 \cdot 7H_2O$
Mg	8.5	8.44	$MgSO_4 \cdot 7H_2O$, $MgCl_2 \cdot 6H_2O$
B	0.023	0.023	$B_4Na_2O_7$
Ba	0.022	0.022	$BaCl_2$
Sr	0.169	0.169	$Sr(NO_3)2$
SO_4	24.7	25.4	$FeSO_4 \cdot 7H_2O$, $MgSO_4 \cdot 7H_2O$, $CuSO_4 \cdot 5H_2O$
K	1.5	1.42	K_2HPO_4, KH_2PO_4, $AlK(SO_4)_2 \cdot 12H_2O$
F	0.63	0.63	NaF
Al	0.03	0.03	$KAl(SO_4)_2 \cdot 12H_2O$
PO_4	1.7	2.03	K_2HPO_4, KH_2PO_4
NO_3	0.37	0.422	$NaNO_3$
Cl	27.3	28.3	$BaCl_2$, $MgCl_2 \cdot 6H_2O$, $CaCl_2$
Ca	32.8	31.6	$CaCl_2$, $Ca(OH)_2$
CO_3	70.62	35.9	$NaHCO_3$
Mo	0.0012	0.00119	$Na_2MoO_4 \cdot 2H_2O$
Cu	0.0058	0.00585	$CuSO_4 \cdot 5H_2O$
Optional: Total organic carbon	1.8	1.2 – 1.4	Tannic acid or humic acid sodium salt
Hardness (calculated)	117	114	$CaCl_2$, $Ca(OH)_2$, $MgSO_4 \cdot 7H_2O$, $MgCl_2 \cdot 6H_2O$
pH	7.3 – 7.8	7.2 – 7.6	HCl, KOH

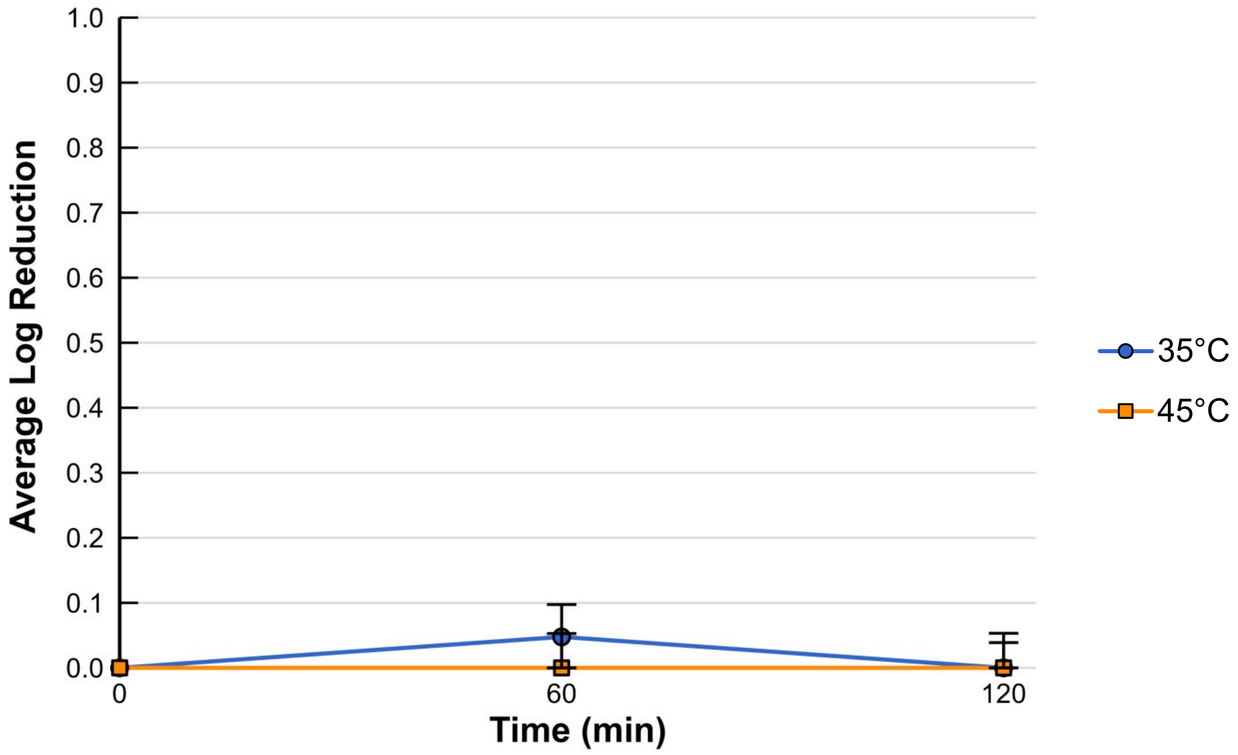

Figure S1. The effect of temperature on *Legionella pneumophila* viability in synthetic tap water at 35°C and 45°C. The initial density of *L. pneumophila* was $7x10^4$ colony forming units per milliliter. Data are presented as mean ± standard error of the mean (n = 3–4).

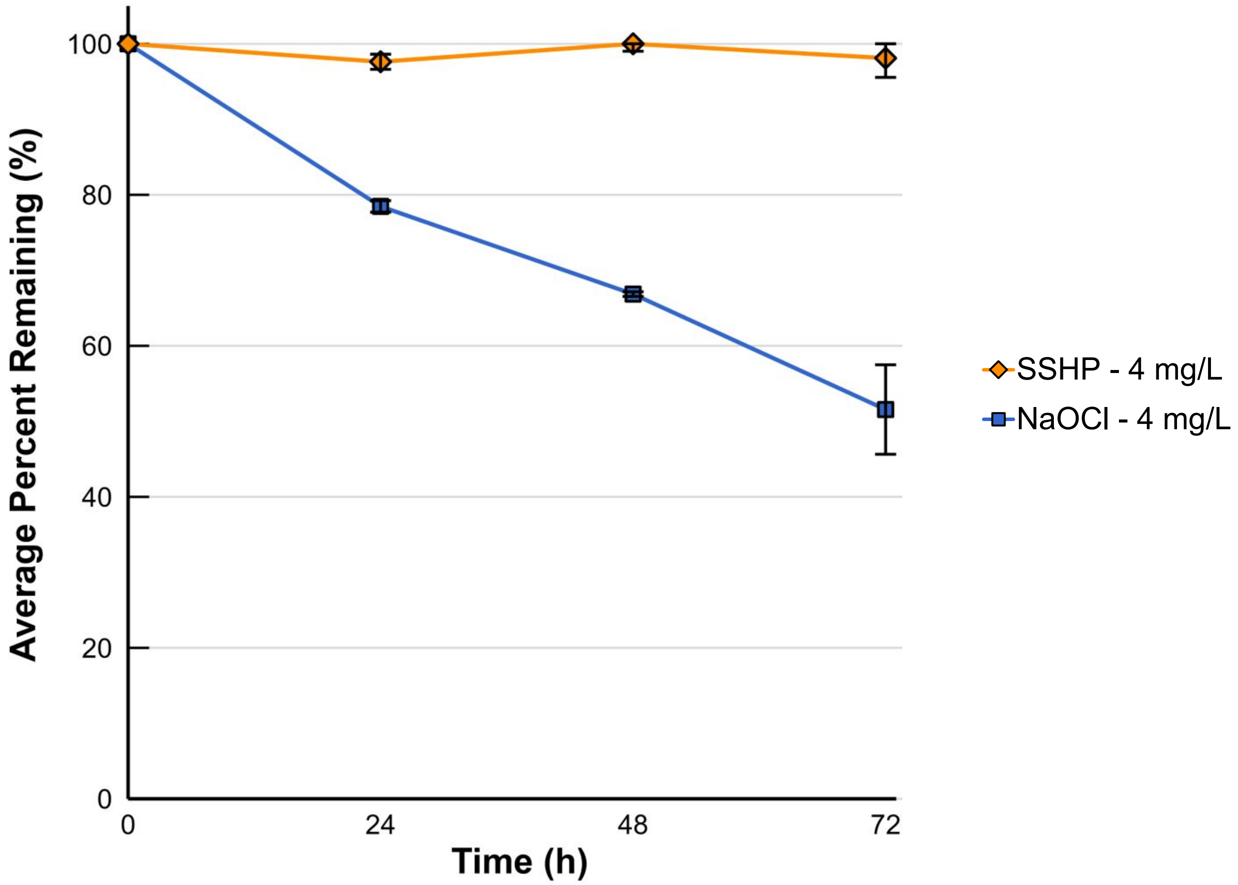

Figure S2. The average remaining percentage of silver-stabilized hydrogen peroxide (SSHP) and sodium hypochlorite (NaOCl; i.e., chlorine) following 72 hours of incubation at 55°C in Milli-Q ultrapure water. Free chlorine was measured for NaOCl, and hydrogen peroxide was measured for SSHP. Data are presented as mean ± standard error of the mean (n = 4).

a)

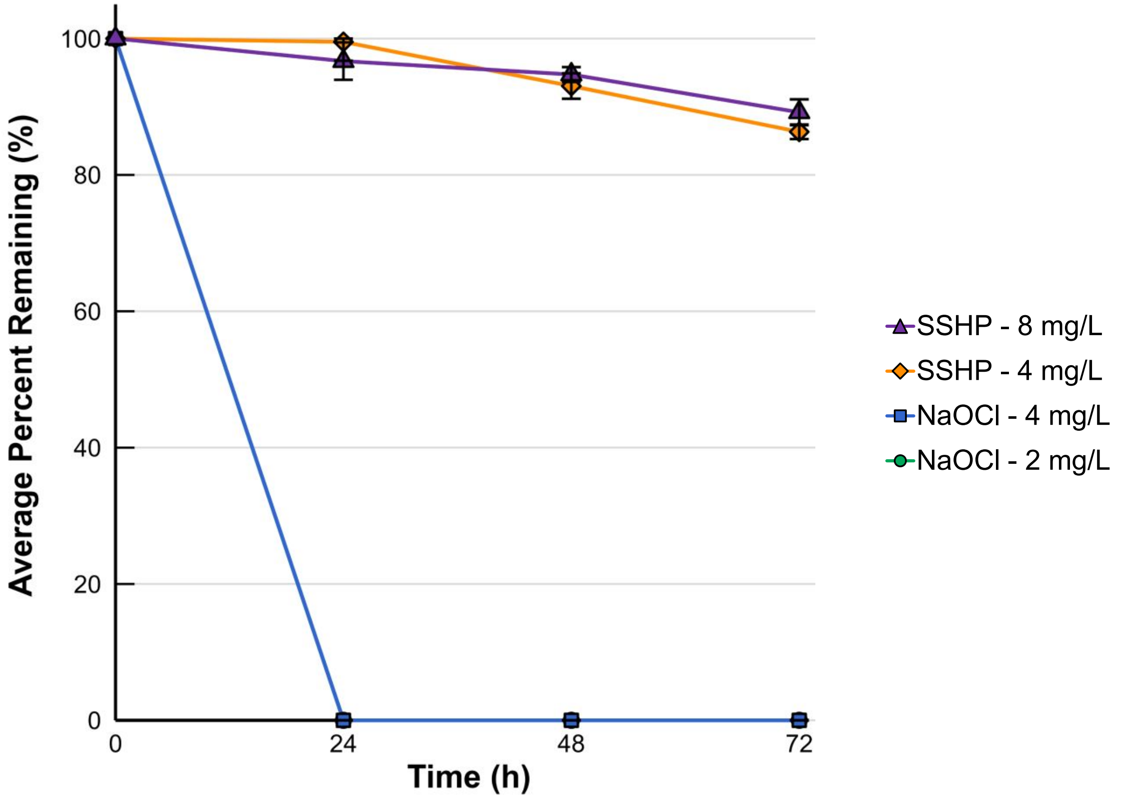
Average Percent Remaining (%)
100
80
60
40
20
0
0
24
48
72
Time (h)
SSHP - 8 mg/L
SSHP - 4 mg/L
NaOCl - 4 mg/L
NaOCl - 2 mg/L

b)

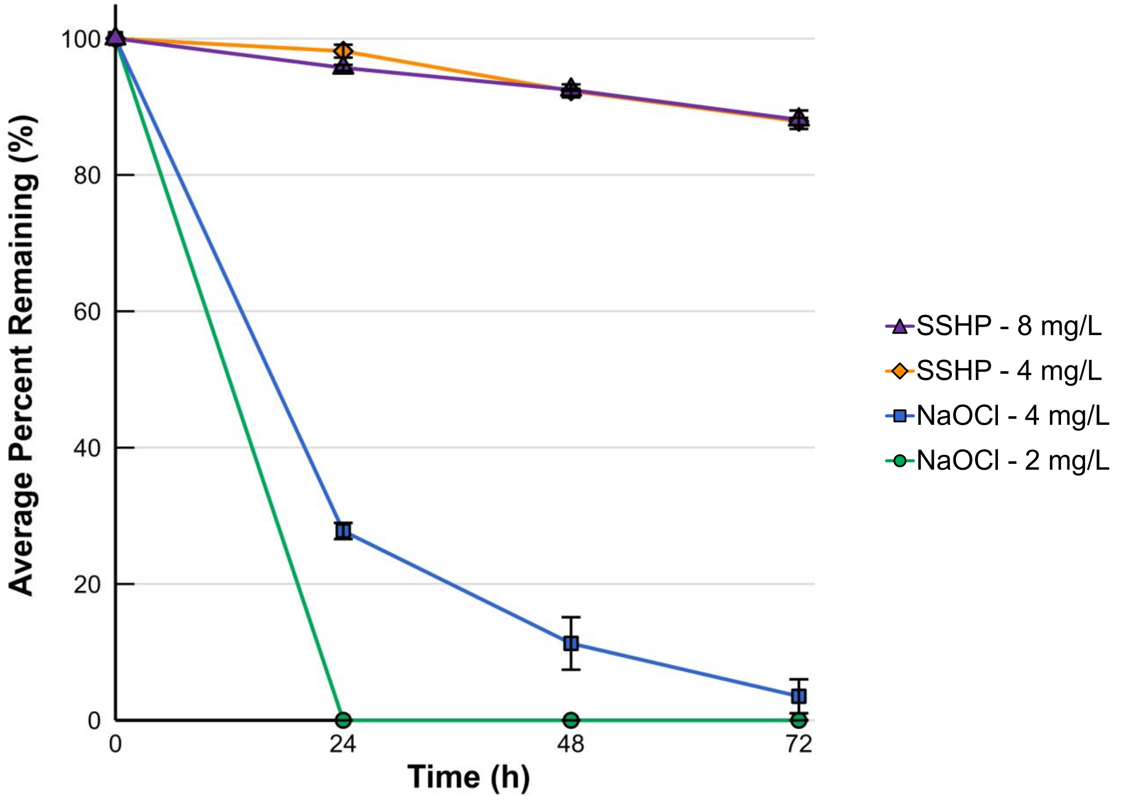
Average Percent Remaining (%)
100
80
60
40
20
0
0
24
48
72
Time (h)
SSHP - 8 mg/L
SSHP - 4 mg/L
NaOCl - 4 mg/L
NaOCl - 2 mg/L

Figure S3. The average remaining percentage of silver-stabilized hydrogen peroxide (SSHP) and sodium hypochlorite (NaOCl; i.e., chlorine) following 72 hours of incubation at 55°C in synthetic tap water containing organics added as contamination. The organics used were **a)** tannic and **b)** humic acid, added to achieve a total organic carbon concentration of 1.4 mg/L. Free chlorine was measured for NaOCl, and hydrogen peroxide was measured for SSHP. Note that in **a)** the lines for 2 and 4 mg/L of NaOCl overlap, as both decayed at the same rate regardless of their starting concentration. Data are presented as mean ± standard error of the mean (n = 3).

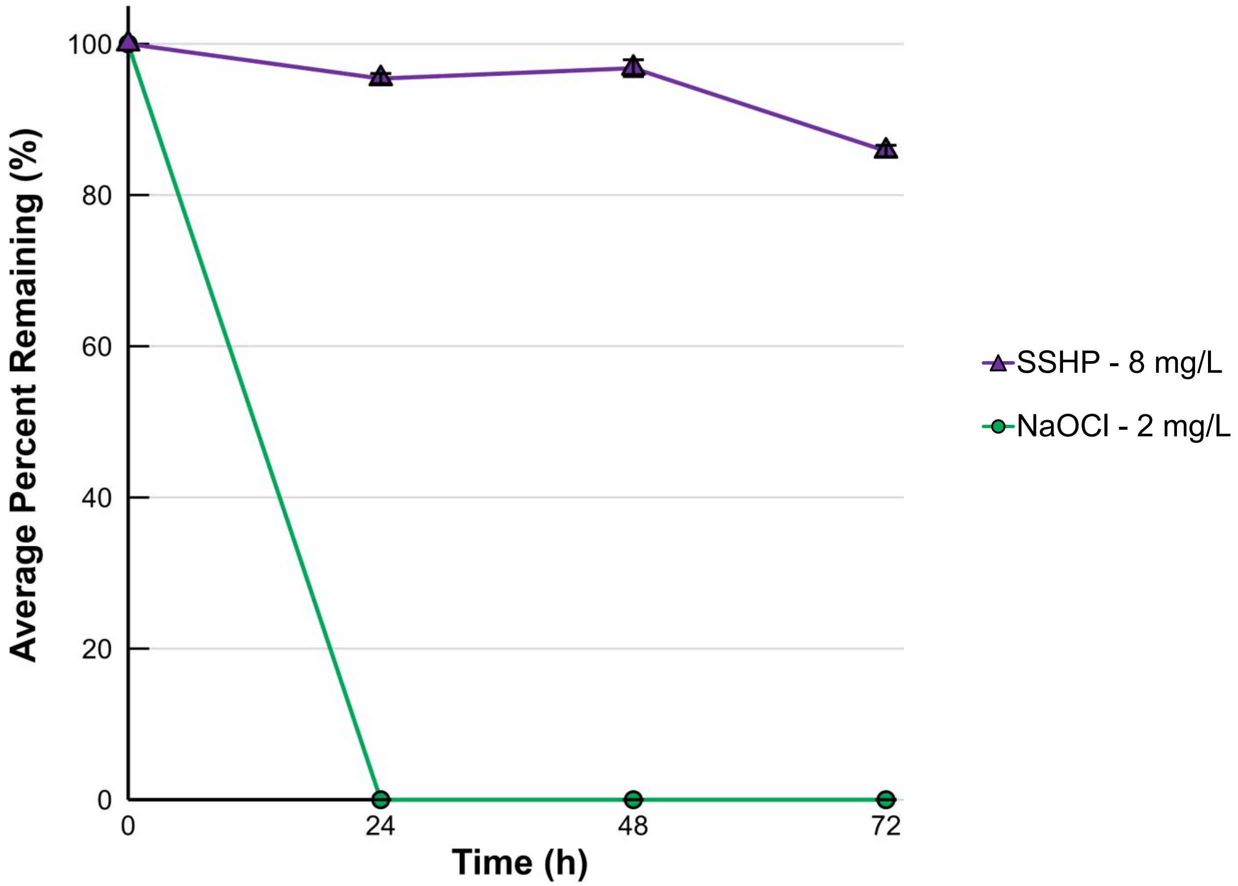

Figure S4. The average remaining percentage of silver-stabilized hydrogen peroxide (SSHP) and sodium hypochlorite (NaOCl; i.e., chlorine) following 72 hours of incubation at 60°C in synthetic tap water supplemented with humic acid (SYTW + HA; 1.2 – 1.4 mg/L total organic carbon). Free chlorine was measured for NaOCl, and hydrogen peroxide was measured for SSHP. The initial SSHP and NaOCl concentrations were 8 and 2 mg/L, respectively. Data are presented as mean ± standard error of the mean (n = 3–4).

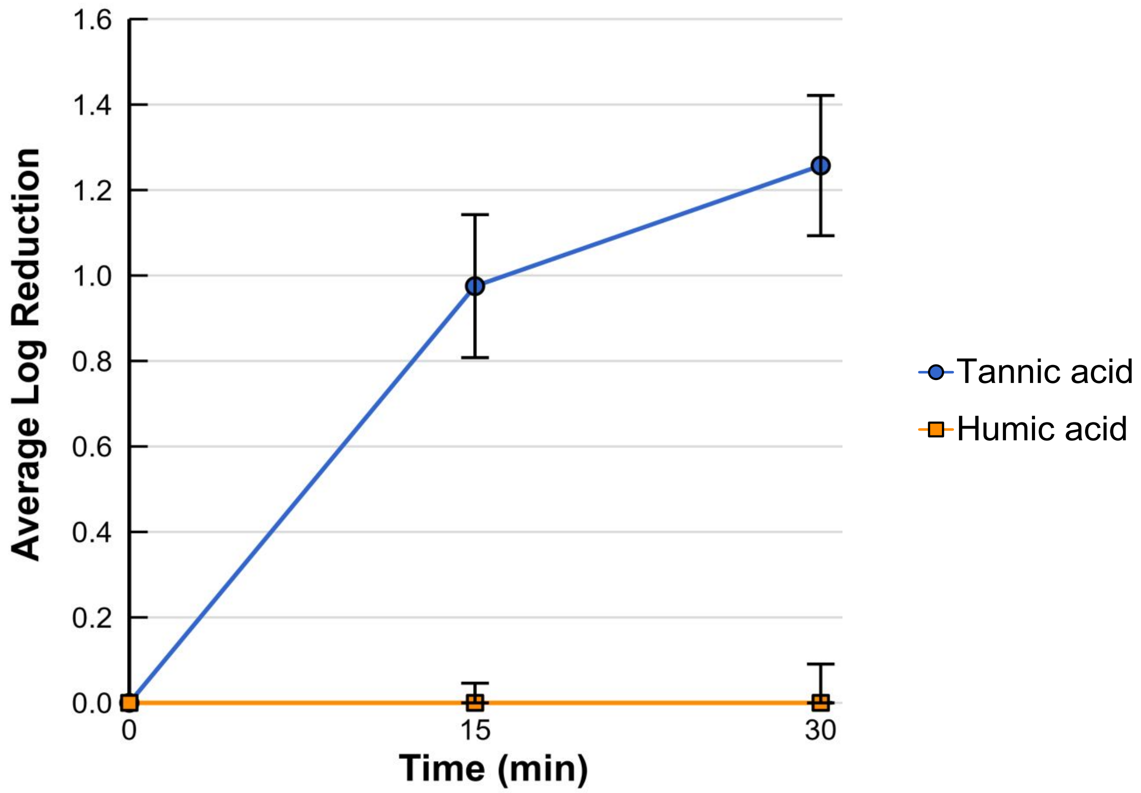

Figure S5. The effect of organic matter type on *Legionella pneumophila* viability in synthetic tap water at 45°C. The synthetic medium was supplemented with either tannic or humic acid (1.2 – 1.4 mg/L total organic carbon). The initial density of *L. pneumophila* was $1x10^5$ colony forming units per milliliter. Data are presented as mean ± standard error of the mean (n = 4).

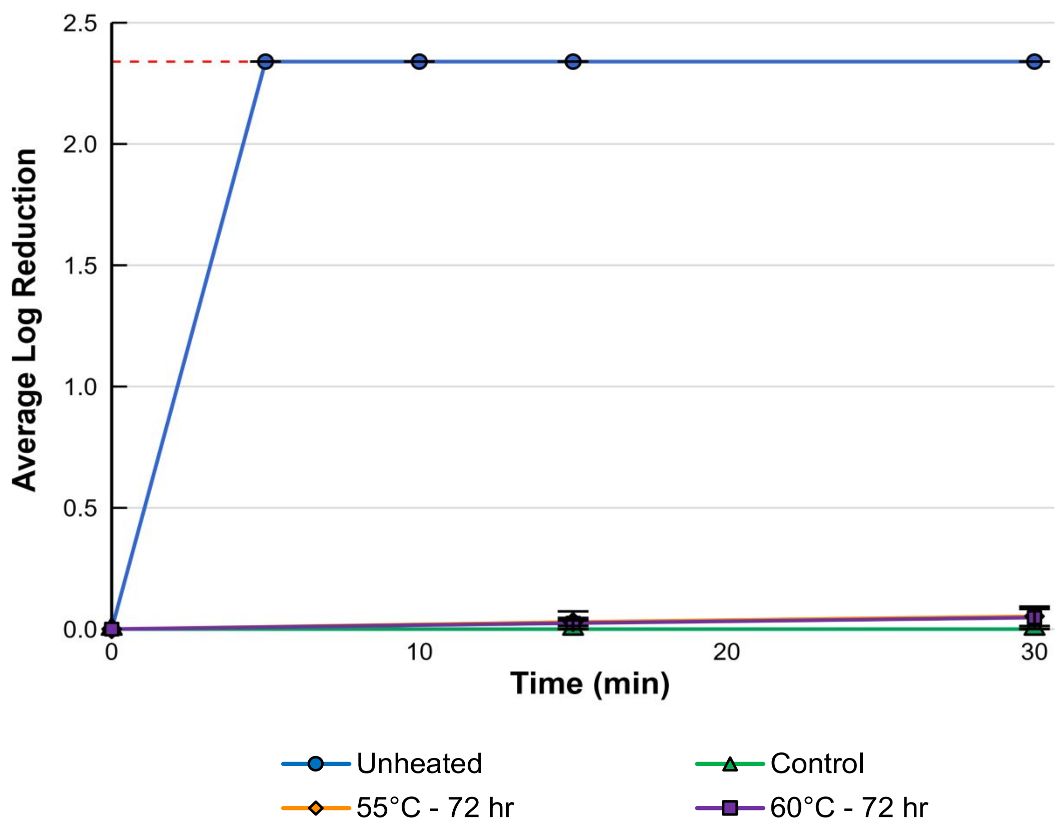

Figure S6. Effect of thermal aging on the biocidal activity of chlorine (2 mg/L) in synthetic tap water supplemented with humic acid (1.2 – 1.4 mg/L total organic carbon). Prior to testing, water was pre-treated by breakpoint chlorination. To simulate prolonged storage in hot water tanks, chlorine solutions were incubated at 55°C for 72 hours or 60°C for 24 hours. Disinfection assays were then performed at 45°C against *Legionella pneumophila* (initial concentration: 1×10^5 colony forming units per millilitre), representing temperatures typical of distal outlets in hot water systems. Freshly prepared chlorine achieved rapid inactivation, reaching the assay's detection limit within 5 minutes. In contrast, thermally aged chlorine showed significantly reduced biocidal activity, with log reductions not significantly different

from the untreated control (p-value > 0.05). The red dashed line indicates the limit of detection. Data are presented as mean ± standard error of the mean (n = 3–5).

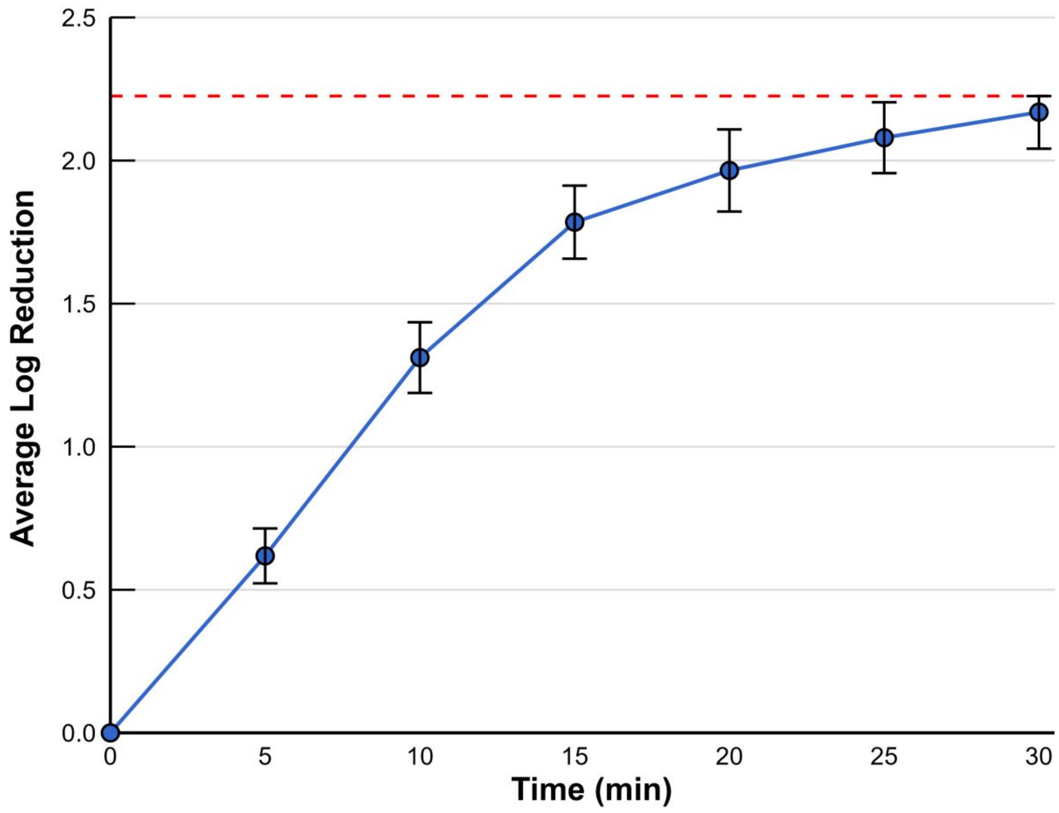

Figure S7. Biocidal activity of freshly prepared 4 mg/L silver-stabilized hydrogen peroxide (SSHP) in synthetic tap water amended with humic acid (1.2–1.4 mg/L total organic carbon). The 4 mg/L SSHP concentration was selected to represent the midpoint of the thermally aged SSHP residuals measured in municipal tap water after 72 hours at 60 °C. Disinfection assays were conducted at 45 °C against *Legionella pneumophila* (initial concentration: 5×10^4 colony forming units per millilitre), representing temperatures typical of distal outlets in hot water systems. The red dashed line denotes the limit of detection. Data are presented as mean ± standard error of the mean (n = 6).
